# Delayed diagnosis of tuberculosis in persons living with HIV in Eastern Europe: associated factors and effect on mortality—a multicentre prospective cohort study

**DOI:** 10.1186/s12879-021-06745-w

**Published:** 2021-10-06

**Authors:** Christian Kraef, Adrian Bentzon, Alexander Panteleev, Alena Skrahina, Natalie Bolokadze, Simona Tetradov, Regina Podlasin, Igor Karpov, Elena Borodulina, Elena Denisova, Inga Azina, Jens Lundgren, Isik Somuncu Johansen, Amanda Mocroft, Daria Podlekareva, Ole Kirk, for the T. B.:H. I. V. Study Group, A. Vassilenko, D. Klimuk, O. Kondratenko, A. Zalutskaya, V. Bondarenko, V. Mitsura, E. Kozorez, O. Tumash, O. Suetnov, D. Paduto, V. Iljina, T. Kummik, K. Mshvidobadze, N. Lanchava, L. Goginashvili, L. Mikiashvili, N. Bablishvili, B. Rozentale, I. Zeltina, I. Janushkevich, I. Caplinskiene, S. Caplinskas, Z. Kancauskiene, A. Wiercinska-Drapalo, M. Thompson, J. Kozlowska, A. Grezesczuk, M. Bura, B. Knysz, M. Inglot, A. Garlicki, J. Loster, D. D uiculescu, A. Rakhmanova, O. Panteleev, A. Yakovlev, A. Kozlov, A. Tyukalova, Y. Vlasova, T. T. rofimov, G. Kyselyova, N. Obel, J. Gerstoft, G. Kronborg, M. C. Payen, K. K abeya, C. Necsoi, F. Dabis, A. Tsaranazy, C. Cazanave, H. Furrer, M. Sagette, M. Rickenbach, D. Sculier, A. Calmy, M. Cavassini, A. Bruno, E. Bernasconi, M. Hoffmann, P. Vernazza, J. Fehr, R. Weber, R. Miller, N. Vora, G. Cooke, S. Mullaney, E. Wilkins, V. George, P. Collini, D. Dockrell, F. Post, L. Campbell, R. Brum, E. Mabonga, P. Saigal, S. Kegg, J. Ainsworth, A. Waters, J. Dhar, K. Ellis, E. Girardi, A. Rianda, V. Galati, C. Pinnetti, C. Tommasi, G. Lapadula, A. Di Biagio, A. Parisini, S. Carbonara, G. Angarano, M. Purgatorio, A. Matteelli, A. Apostoli, J. M. Miro, C. Manzardo, C. Ligero, J. Gonzalez, Jose A. Martinez-Martinez, F. Sanchez, H. Knobel, M. Salvadó, J. L. Lopez-Colomes, X. Martínez-Lacasa, E. Cuchí, V. Falcó, A. Curran, M. T. Tortola, I. Ocaña, R. Vidal, M. A. Sambeat, V. Pomar, P. Coll, D. Pozamczer, M. Saumoy, F. Alcaide, J. Caylà, A. Moreno, J. P. Millet, A. Orcau, L. Fina, A. Romero, L. L. Roldan, J. A. Iribarren, M. Ibarguren, S. Moreno, A. González, P. Miralles, T. Aldámiz-Echevarría, M. Losso, J. Toibaro, L. Gambardella, J. Toibaro, L. Moreno Macias, E. Warley, S. Tavella, O. Garcia Messina, O. Gear, H. Laplume, C. Marson, J. Contarelia, M. Michaan, P. Scapellato, B. Bartoletti, D. Palmero, C. Elias, C. Cortes, B. Crabtree, J. L. Mosqueda Gomez, J. A. Villanueva, L. A. Gonzalez Hernandez, F. Badial

**Affiliations:** 1 CHIP (Centre of Excellence for Health, Immunity and Infections), Rigshospitalet, University of Copenhagen, Copenhagen, Denmark; 2 Department of Infectious Diseases, The Heart Centre, Rigshospitalet, University of Copenhagen, Copenhagen, Denmark; 3 City TB Dispensary, St. Petersburg, Russia; 4 Republican Scientific and Practical Center for Pulmonology and Tuberculosis, Minsk, Belarus; 5 Infectious Diseases, AIDS and Clinical Immunology Research Center, Tbilisi, Georgia; 6 Dr Victor Babes’ Hospital of Tropical and Infectious Diseases, Bucharest AND ‘Carol Davila’ University of Medicine and Pharmacy, Bucharest, Romania; 7 Wojewodski Szpital Zakanzy/Medical University of Warsaw, Warsaw, Poland; 8 Department of Infectious Disease, Belarusian State Medical University, Minsk, Belarus; 9 Samara State Medical University of the Ministry of Healthcare of the Russian Federation, Samara, Russia; 10 Botkin Hospital of Infectious Disease, St. Petersburg, Russia; 11 Riga East University Hospital, Latvian Centre of Infectious Diseases, Riga, Latvia; 12 Research Unit for Infectious Diseases, Odense University Hospital, University of Southern, Odense, Denmark; 13 Centre for Clinical Research, Epidemiology, Modelling and Evaluation (CREME), Institute for Global Health, UCL, London, UK; 14 Heidelberg Institute of Global Health, University of Heidelberg, Heidelberg, Germany

## Abstract

**Background:**

Early diagnosis of tuberculosis (TB) is important to reduce transmission, morbidity and mortality in people living with HIV (PLWH).

**Methods:**

PLWH with a diagnosis of TB were enrolled from HIV and TB clinics in Eastern Europe and followed until 24 months. Delayed diagnosis was defined as duration of TB symptoms (cough, weight-loss or fever) for ≥ 1 month before TB diagnosis. Risk factors for delayed TB diagnosis were assessed using multivariable logistic regression. The effect of delayed diagnosis on mortality was assessed using Kaplan–Meier estimates and Cox models.

**Findings:**

480/740 patients (64.9%; 95% CI 61.3–68.3%) experienced a delayed diagnosis. Age ≥ 50 years (vs. < 50 years, aOR = 2.51; 1.18–5.32; p = 0.016), injecting drug use (IDU) (vs. non-IDU aOR = 1.66; 1.21–2.29; p = 0.002), being ART naïve (aOR = 1.77; 1.24–2.54; p = 0.002), disseminated TB (vs. pulmonary TB, aOR = 1.56, 1.10–2.19, p = 0.012), and presenting with weight loss (vs. no weight loss, aOR = 1.63; 1.18–2.24; p = 0.003) were associated with delayed diagnosis. PLWH with a delayed diagnosis were at 36% increased risk of death (hazard ratio = 1.36; 1.04–1.77; p = 0.023, adjusted hazard ratio 1.27; 0.95–1.70; p = 0.103).

**Conclusion:**

Nearly two thirds of PLWH with TB in Eastern Europe had a delayed TB diagnosis, in particular those of older age, people who inject drugs, ART naïve, with disseminated disease, and presenting with weight loss. Patients with delayed TB diagnosis were subsequently at higher risk of death in unadjusted analysis. There is a need for optimisation of the current TB diagnostic cascade and HIV care in PLWH in Eastern Europe.

**Supplementary Information:**

The online version contains supplementary material available at 10.1186/s12879-021-06745-w.

## Introduction

Globally, about 10 million people were diagnosed with tuberculosis (TB) in 2019 [1]. Among those, 8.2% were people living with the human immunodeficiency virus (PLWH) [1]. Among PLWH, TB is the most frequent co-infection, cause for hospitalization and cause of death, responsible for about 208,000 AIDS-related deaths worldwide in 2019 [1, 2]. One major challenge to improve survival for TB patients is to ensure timely diagnosis of TB [3]. In patients mono-infected with TB a recent systematic review of delay in diagnosis of pulmonary TB in low- and middle income countries found that 42% of TB patients had a delay between symptom onset and TB diagnosis of at least a month [4]. A systematic review of post-mortem studies from Africa, Asia and the Americas found TB as the cause of death in 37.2% of PLWH, of which 45.8% remained undiagnosed at the point of death [3]. Delayed diagnosis of TB has repeatedly been shown to lead to more severe disease presentation, and increases morbidity and mortality significantly [5–11]. Furthermore, delayed TB diagnosis increases the risk for TB community transmission [6, 12].

In consequence, one component of the World Health Organization’s End TB strategy to reduce TB deaths by 75% and TB incidence by 50% in the general population by the year 2025 is ensuring early diagnosis of TB [13]. To this end, the strategy identifies amongst others the mapping of high-risk groups and the implementation of carefully planned systematic screening for active disease among identified high-risk groups as important facilitators of early diagnosis [13]. One high-risk group are PLWH, in whom the effect of delayed diagnosis on morbidity and mortality is even more distinct compared to those mono-infected with TB [14–16].

HIV/TB-coinfections continue to be a major challenge in many Eastern European countries [17]. In a large clinical cohort of patients with HIV/TB across Eastern Europe, 1-year mortality was 27%, with the majority of those deaths (79%) directly attributable to TB [17]. That is a risk of death nearly four-times higher than that in PLWH with TB from western Europe and Latin America [17].

However, to the best of our knowledge, the contribution of delayed TB diagnosis to morbidity and mortality and its associated factors in PLWH have not been investigated in an Eastern European setting. The objective of this study is to describe the prevalence of delayed diagnosis, analyze factors associated with delayed diagnosis and to quantify the effect of delayed diagnosis on survival in PLWH.

## Methods

### Study design, population and sample

The data for this analysis were drawn form an international prospective observational cohort study (TB:HIV study). Details of the study have previously been published [17]. Study protocols and forms are available at www.chip.dk. Only patients included at Eastern European study sites were included in the current analysis. The TB:HIV study enrolled consecutive HIV-patients with TB diagnosis aged 16 years or older from 21 HIV and TB clinics in nine Eastern European countries (Belarus, Estonia, Georgia, Latvia, Lithuania, Poland, Romania, Russia and Ukraine) between Jan 1, 2011 and Dec 31, 2013 [17]. All methods were carried out in accordance with relevant guidelines and regulations. All participating clinics obtained ethical approval in accordance with local rules and legislations. A list of IEC/IRB from each participating center is provided in Appendix. Informed consent was obtained from all participants. The study was performed in accordance with the STROBE guidelines for observational studies [18].

### Variables, inclusion and exclusion criteria

Explanatory variables include demography data, previous TB-disease, type of TB diagnosis, clinical presentation of current TB, HIV characteristics including CD4 cell counts and HIV-RNA measurements, antiretroviral therapy (ART) history and AIDS defining diagnoses. Variables with missing information on treatment with cotrimoxazole, chest x-ray, HBsAg, Anti-HCV and TB risk factors (alcohol, previous TB, imprisonment) were assumed as absent. Those with missing information on CD4 count were included as a separate category. For drug-susceptibility testing and determination of MDR-TB, if some information on drug-susceptibility was present, but missing for a specific drug *M. tuberculosis* was assumed to be susceptible to that specific drug, as done previously [19].

To evaluate the delayed diagnosis, we used the variable “symptom duration” prior to diagnosis that was captured as a categorical variable (< 1 month, 1–3 months and > 3 months) in the original enrollment form by the enrolling health care worker based on self-reporting by the PLWH. For the purpose of this study, we merged the categories to create a binary variable (≥ 1 months versus < 1 months).

**Table:** Box 1. Study definitions

Region	Eastern Europe	Belarus, Estonia, Georgia, Latvia, Lithuania, Poland, Romania, Ukraine, Russia
Delayed diagnosis		Delayed diagnosis was defined as duration of TB symptoms (cough, weight-loss or fever) for ≥ 1 month before TB diagnosis
TB diagnosis	Definite	Positive culture or PCR (Xpert) for Mtb (in sputum smear or other specimen)
	Probable	Acid fast bacilli or granulomatous inflammation in sputum smear or tissue biopsy and other specimens (e.g., CSF, Pleural fluid)
	Presumptive	A patient who presents with symptoms or signs suggestive of TB where TB treatment is initiated and not subsequently stopped because the TB diagnosis was ruled out
TB location	Pulmonary	TB localised to the lungs, larynx, or tracheobronchial tree
	Extrapulmonary	just one extra-pulmonary site was identified
	Disseminated	Either of the following: (i) TB documented in at least two organ systems (one of which could be lungs) (ii) miliary TB, or (iii) isolation of Mtb from blood or bone marrow
TB drug resistance	MDR	Mtb resistant to both rifamycin and isoniazid
AIDS		Clinical AIDS criteria (ref: 1993 Revised Classification System for HIV Infection and Expanded Surveillance Case Definition for AIDS Among Adolescents and Adults)

### Data analysis

Descriptive statistics were used to present data as proportions, medians and interquartile ranges. The Chi-squared and Fisher’s exact test were used to compare categorical variables between groups, while the Kruskal–Wallis test was used to compare continuous variables.

To identify risk factors for delayed diagnosis we first conducted a bivariate analysis of potential risk factors. Those with a p ≤ 0.10 were included in the multivariable logistic regression model.

For the survival analysis baseline was defined as the date of TB treatment initiation. All follow-up was censored at 24 months. Overall survival comparing the group with delayed and non-delayed diagnosis was assessed using the Kaplan–Meier (KM) method and the log-rank test. We used Cox proportional hazards regression models to estimate hazard ratios (HRs) and 95% confidence intervals (CIs) of the effect of delayed diagnosis on mortality. Variables with a p-value ≤ 0.10 were included in the multivariate model to assess the hazard ratio (HR) and the Cox-model was stratified by the clinical center. Based on the Kaplan–Maier survival analysis, we decided to stratify the Cox-regression model at 2 months follow-up time (0–2 months and 2–24 months). The proportional hazard assumption was tested based on Schoenfeld residuals. Stata IC 15.1 was used for statistical analysis.

## Results

In the TB:HIV cohort 825 PLWH from Eastern Europe were eligible for the analysis, of those 85 (10.3%) were excluded because of missing data on symptom duration.

A total of 740 patients from Eastern Europe were included in the current analysis. The analysis included 260 (35.1%) patients with a delay of less than 1 month and 480 (64.9%) patients with a delay of at least 1 month, 380 (51.4%) had a delay of 1–3 months, and 100 (13.5%) of > 3 months.

### Factors associated with delayed diagnosis

Baseline characteristics of the TB:HIV patients in Eastern Europe stratified by delayed diagnosis, and factors associated with delayed diagnosis are presented in Table 1. In the multivariable analysis age ≥ 50 years (vs. < 50 years, aOR 2.51, 95% CI 1.18–5.32, p = 0.016), Injecting Drug Use (IDU) (vs. non-IDU aOR 1.66, 95% CI 1.21–2.29, p = 0.002), being ART treatment naïve at TB diagnosis (vs. any ART before TB diagnosis, aOR 1.77, 95% CI 1.24–2.54, p = 0.002), disseminated TB (vs. pulmonary TB, aOR 1.56, 95% CI 1.10–2.19, p = 0.012), and weight loss (vs. no weight loss, aOR 1.63, 95% CI 1.18–2.24, p = 0.003) were associated with delayed diagnosis (≥ 1 month). Conversely, a previous TB diagnosis (vs. never diagnosed with TB, aOR 0.60, 95% CI 0.38–0.95, p = 0.029) was associated with earlier diagnosis.

**Table 1:** Baseline characteristics of the TB:HIV patients in Eastern Europe stratified by delayed diagnosis and factors associated with delayed diagnosis

	Total	Delayed diagnosis		OR (95% CI)	p-value	Adjusted OR (95% CI)	p-value
		< 1 month	≥ 1 month				
	n = 740	n = 260	n = 480				
Gender							
Male	563 (76.1)	200 (76.9)	363 (75.6)	0.93 (0.65–1.33)	0.69		
Age							
Age 16–49	694 (93.8)	250 (96.2)	444 (92.5)	Ref	Ref	Ref	Ref
Age ≥ 50	46 (6.2)	10 (3.8)	36 (7.5)	2.62 (1.21–5.66)	0.01	2.51 (1.18–5.32)	0.016
Exposure group (HIV)							
MSM (yes vs no)	10 (1.4)	5 (1.9)	5 (1.0)	0.54 (0.15–1.87)	0.32		
IDU (yes vs no)	422 (57.0)	132 (50.8)	290 (60.4)	1.48 (1.09–2.00)	0.01	1.66 (1.21–2.29)	0.002
Heterosexual (yes vs no)	183 (24.7)	72 (27.7)	111 (23.1)	0.79 (0.56–1.11)	0.17		
Known HIV at TB diagnosis	668 (90.3)	235 (90.4)	433 (90.2)	0.98 (0.59–1.63)	0.94		
Treatment history (HIV)							
ART naïve at TB diagnosis^a^	558 (75.4)	176 (67.7)	382 (79.6)	1.86 (1.32–2.63)	0.0001	1.77 (1.24–2.54)	0.002
ART use at TB diagnosis^a^	132 (17.8)	59 (22.7)	73 (15.2)	0.61 (0.42 –0.9)	0.011	Omitted^d^	
Cotrimoxazole at TB diagnosis	273 (36.9)	84 (32.3)	189 (39.4)	1.36 (0.99–1.87)	0.057	1.25 (0.89–1.74)	0.197
CD4-cell count/mm^3^°							
0–199/mm^3^	440 (59.5)	151 (58.1)	289 (70.8)	1.06 (0.74–1.52)	0.745		
≥ 200	185 (25.0)	66 (25.4)	119 (29.2)	Ref	Ref		
Missing CD4	115 (15.5)	43 (37.4)	72 (62.6)	0.93 (0.57–1.51)	0.764		
Prior AIDS	177 (23.9)	66 (25.4)	111 (23.1)	0.88 (0.62–1.26)	0.49		
Previous TB							
Yes vs no	99 (13.4)	46 (17.7)	53 (11.0)	0.58 (0.38–0.89)	0.011	0.60 (0.38–0.95)	0.029
TB risk factor							
Alcohol abuse (yes vs no)	186 (25.1)	72 (27.7)	114 (23.8)	0.81 (0.58–1.15)	0.24		
Recent TB in family (yes vs no)	53 (7.2)	17 (6.5)	36 (7.5)	1.16 (0.64–2.11)	0.63		
Imprisonment within 2 years (yes vs no)	143 (19.3)	47 (18.1)	96 (20.0)	1.13 (0.77–1.67)	0.53		
Clinical presentation of TB							
Pulmonary (including trachea and larynx)	239 (32.3)	101 (38.9)	138 (28.8)	Ref	Ref	Ref	Ref
Extrapulmonary	54 (7.3)	23 (8.9)	31 (6.5)	0.99 (0.54–1.79)	0.964	0.83 (0.44–1.55)	0.553
Disseminated	447 (60.4)	136 (52.3)	311 (64.8)	1.67 (1.21–2.32)	0.002	1.56 (1.10–2.19)	0.012
Localisations TB was found (non-exclusive)							
Pulmonary (including trachea and larynx)	654 (88.4)	227 (87.3)	427 (89.0)	1.17 (0.74–1.86)	0.504	Omitted^e^	
Pleural	92 (12.4)	32 (12.3)	60 (12.5)	1.02 (0.64–1.61)	0.94	Omitted^e^	
Lymphatic	309 (41.8)	76 (29.2)	233 (48.5)	2.28 (1.65–3.17)	0.0001	Omitted^e^	
Bone and/or joint	13 (1.8)	2 (0.8)	11 (2.3)	3.03 (0.66–13.8)	0.155	Omitted^e^	
Genitourinary	37 (5.0)	5 (1.9)	32 (6.7)	3.64 (1.39–9.52)	0.004	Omitted^e^	
Meningeal and other central nervous system	74 (10.0)	28 (10.8)	46 (9.6)	0.88 (0.53–1.44)	0.608	Omitted^e^	
Gastro-intestinal	55 (7.4)	16 (6.2)	39 (8.1)	1.35 (0.74–2.46)	0.329	Omitted^e^	
TB symptoms							
Cough (yes vs no)	501 (67.7)	188 (72.3)	313 (65.2)	0.72 (0.52–0.99)	0.049	0.72 (0.51–1.02)	0.061
Fever (yes vs no)	636 (86.0)	227 (87.3)	409 (85.2)	0.84 (0.54–1.31)	0.433		
Weight loss (yes vs no)	445 (60.1)	133 (51.2)	312 (65.0)	1.77 (1.3–2.42)	0.0001	1.63 (1.18–2.24)	0.003
Number of symptoms^c^							
None	20 (2.7)	6 (2.3)	14 (2.9)	Ref	Ref		
One symptom	133 (18.0)	50 (19.2)	83 (17.3)	0.71 (0.26–1.97)	0.512		
Two symptoms	312 (42.2)	114 (43.9)	198 (41.3)	0.74 (0.28–2.0)	0.556		
Three symptoms	275 (37.2)	90 (34.6)	185 (38.5)	0.88 (0.33–2.37)	0.802		
Screening chest X-ray before current episode							
Yes vs no	338 (45.7)	115 (44.2)	223 (46.5)	1.09 (0.81–1.48)	0.56		
Rifampicin resistance							
Yes vs no	95 (38.3)	34 (35.8)	61 (39.9)	1.19 (0.7–2.0)	0.52		
MDR-TB							
Yes	88 (11.9)	31 (11.9)	57 (11.9)	1.22 (0.71–2.11)	0.478		
No	148 (20.0)	59 (22.7)	89 (18.5)	Ref	Ref		
Missing drug susceptibility testing	504 (68.1)	170 (65.4)	334 (69.6)	1.30 (1.09–1.90)	0.170		
Diagnosis							
Definite	333 (45.0)	123 (47.3)	210 (43.8)	Ref	Ref		
Probable	76 (10.3)	24 (9.2)	52 (10.8)	1.27 (0.75–2.16)	0.380		
Presumptive	331 (44.7)	113 (43.5)	218 (45.4)	1.13 (0.82–1.55)	0.451		
Hepatitis B							
HbsAg at baseline^b^	44 (6.0)	17 (6.5)	27 (5.6)	0.85 (0.46–1.59)	0.616		
Hepatitis C							
Anti-HCV antibodies^b^	405 (54.7)	134 (51.5)	271 (56.5)	1.21 (0.9–1.65)	0.199		
Continuous variables							
Weight (kg)							
Baseline	60 (53–68)	63 (55–68.5)	60 (53–68)	0.99 (0.97–1.01)	0.255		
HIV-RNA (log10) copies/ml							
Baseline	5.3 (4.5–5.8)	5.2 (4.3–5.7)	5.3 (4.6–5.8)	1.12 (0.96–1.32)	0.158		

Adjusted for age, IDU, ART treatment naivety, cotrimoxazole treatment, previous TB, clinical TB presentation and TB symptoms cough and weight loss

*OR* odds ratio, *CI* confidence interval, *MSM* Men Who Have Sex with Men, *IDU* injecting drug use

^a^Those not included in the categories “ART naïve at TB diagnosis” and”ART use at TB diagnosis” have previously received ART and interrupted their treatment

^b^Those with missing information for chest x-ray (n = 278), HBsAg (n = 215), Anti-HCV (n = 220) assumed negative

^c^Index based on symptoms cough, fever, weight loss

^d^Omitted because of multicollinearity with Treatment Naivety (correlation co-efficient 0.7817)

^e^Omitted in model as part of extra-pulmonary/disseminated

In an analysis of the organ systems affected, i.e. organs where TB was identified during diagnostic work-up (non-exclusive/several sites possible in the same patient) genitourinary (OR 3.64; 95% CI 1.39–9.52; p = 0.006) and lymphatic TB (OR 2.28; 95% CI 1.65–3.17; p = 0.001) were associated with delayed diagnosis, whereas pulmonary TB was not (OR 1.17; 95% CI 0.74–1.86, p = 0.504).

### Survival analysis

**Fig. 1:**
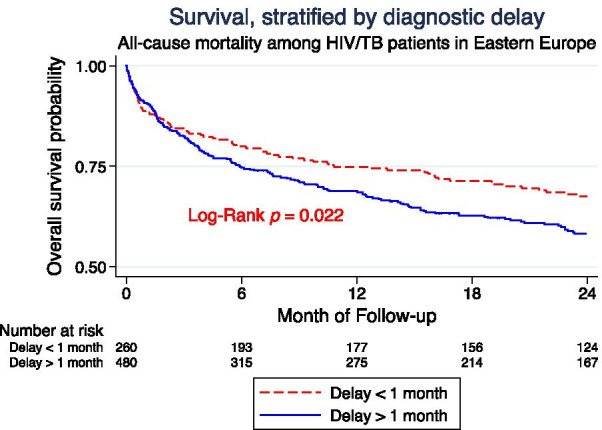
Kaplan–Meier survival estimates by diagnostic delay (< 1 month versus ≥ 1 month)

Survival over 24 months, stratified by delayed diagnosis, is presented in the Kaplan–Maier analysis in Fig. 1. Estimates of all-cause mortality at 24 months were 262 (35.4%), 80 (30.8%) in the early diagnosis group and 182 (37.9%) in the late diagnosis group. In the first 2 months of follow-up, the all-cause mortality rate did not differ between PLWH with and without delayed diagnosis, (100/100-person-years of follow-up (PYFU) (95% CI 78–127) v. 91/100 PYFY, respectively, log-rank test p = 0.566. For the follow-up time from 2 to 24 months all-cause mortality rates were 23/100 PYFU (95% CI 19–28) and 14/100 PYFU (95% CI 11–19), respectively, log-rank test p = 0.011.

In the unadjusted Cox proportional hazard models for all-cause mortality with 24 months follow-up, PLWH with a delayed TB diagnosis were at 36% increased risk of death (hazard ratio 1.36; 95% CI 1.04–1.77; p = 0.023). In the adjusted analysis, the estimate decreases only slightly (adjusted hazard ratio 1.27; 95% CI 0.95–1.70; p = 0.103) (Table 2). At the same time, there is a clear association between increased risk of death and male sex, low or missing CD4 cell count, prior AIDS, disseminated TB disease, weight loss and MDR-TB (Table 2). Stratifying the Cox proportional hazards model for all-cause mortality at 0–2 months and 2–24 months follow-up according to the appearance of the survival curves, no effect of delayed diagnosis on risk of death was observed for the first 2 months (aHR 1.09; 95% CI 0.69–1.71; p = 0.723), while the period 2–24 months seems to explain the observed overall effect (aHR 1.36; 95% CI 0.93–1.99; p = 0.119) (Additional file 1: Tables S1 and S2).

**Table 2:** Cox-regression model for survival in Eastern Europe, crude and adjusted hazard ratios (24 months follow-up)

	Total (n = 740)	Crude HR (95% CI)	p-value	Adjusted HR (95% CI)^a^	p-value
Delayed diagnosis	≥ 1 month (n = 480)	1.36 (1.04–1.77)	0.023	1.27 (0.95–1.70)	0.103
Gender	Male (n = 563)	1.34 (1.02–1.76)	0.032	1.38 (1.04–1.83)	0.026
Age	Age 18–49 (n = 694)	Ref	Ref		
	Age ≥ 50 (n = 46)	0.92 (0.55–1.52)	0.741		
Exposure group (HIV)	MSM (n = 10)	0.49 (0.12–1.95)	0.309		
	IDU (n = 422)	1.19 (0.93–1.52)	0.173		
	Heterosexual (n = 183)	0.84 (0.63–1.13)	0.255		
Known HIV positive	At baseline (n = 668)	1.55 (0.96–2.50)	0.074	1.43 (0.84–2.42)	0.185
Treatment history (HIV)	Naïve at TB diagnosis^c^ (n = 558)	1.04 (0.79–1.38)	0.766		
	ART at baseline^c^ (n = 132)	0.96 (0.70–1.31)	0.795		
	Cotrimoxazole at baseline (n = 273)	1.22 (0.96–1.56)	0.110		
CD4-cell count/mm^3^°	0–199/mm^3^ (n = 440)	2.17 (1.53–3.09)	0.001	1.84 (1.27–2.68)	0.001
	≥ 200/mm^3^ (n = 185)	Ref	Ref	Ref	Ref
	Missing CD4 (n = 115)	2.17 (1.41–3.33)	0.001	2.32 (1.47–3.66)	0.001
Prior AIDS	Yes (n = 177)	1.87 (1.45–2.41)	0.001	1.69 (1.27–2.24)	0.001
Previous TB	Yes (n = 99)	0.97 (0.68–1.39)	0.880		
TB risk factor	Alcohol abuse (n = 186)	1.32 (1.01–1.73)	0.041	1.24 (0.92–1.68)	0.158
	Recent TB in family (n = 53)	0.84 (0.50–1.42)	0.524		
	Prison within the last 2 years (n = 143)	1.11 (0.82–1.51)	0.493		
Clinical presentation of TB	Pulmonary (n = 239)	Ref	Ref	Ref	Ref
	Extrapulmonary (n = 54)	0.98 (0.51–1.88)	0.957	0.86 (0.44–1.68)	0.657
	Disseminated (n = 447)	2.31 (1.71–3.11)	0.001	1.93 (1.38–2.69)	0.001
TB symptoms at diagnosis	Cough (n = 501)	0.95 (0.74–1.23)	0.708		
	Fever (n = 636)	1.59 (1.07–2.37)	0.022	1.41 (0.91–2.19)	0.124
	Weight loss (n = 445)	1.70 (1.31–2.21)	0.001	1.41 (1.05–1.88)	0.022
Number of symptoms§	None (n = 20)	Ref	Ref		
	One symptom (n = 133)	0.89 (0.38–2.11)	0.793		
	Two symptoms (n = 312)	1.12 (0.49–2.56)	0.783		
	Three symptoms (n = 275)	1.58 (0.69–3.58)	0.277		
Screening chest X-ray in past^d^	Yes (n = 338)	1.30 (1.02–1.66)	0.034	1.27 (0.93–1.74)	0.133
Rifampicin resistance	Yes (n = 95)	1.53 (1.12–2.10)	0.007	Omitted^b^	
MDR-TB	Yes (n = 88)	2.61 (1.68–4.05)	0.001	2.06 (1.06–4.01)	0.033
	No (n = 148)	Ref	Ref	Ref	
	Missing (n = 504)	1.84 (1.28–2.64)	0.001	1.32 (0.68–2.57)	0.407
Diagnosis	Definitive (n = 333)	Ref	Ref	Ref	Ref
	Probable (n = 76)	1.43 (0.97–2.11)	0.074	0.99 (0.61–1.59)	0.955
	Presumptive (n = 331)	1.03 (0.80–1.34)	0.795	0.95 (0.67–1.35)	0.764
Hepatis B	HBsAg positive (n = 44)^d^	0.95 (0.56–1.60)	0.843		
Hepatitis C	Anti-HCV positive (n = 405)^d^	1.20 (0.94–1.53)	0.145		
Drug-susceptibility testing	At baseline (n = 236)	0.84 (0.65–1.08)	0.175		
Treatment with RHZ	At baseline (n = 591)	0.81 (0.59–1.09)	0.157		
Treatment with at least three active drugs	At baseline (n = 132)	0.64 (0.46–0.91)	0.014	0.81 (0.52–1.25)	0.344

*HR* hazard ratio, *CI* confidence interval, *MSM* Men Who Have Sex with Men, *IDU* injecting drug use

Global Test Proportional Hazard Assumption p = 0.3689, individually all > 0.05

^a^Adjusted for diagnostic delay, gender, known HIV positive, CD4 cell count, prior aids, tb risk factor alcohol, clinical presentation (all), Tb symptoms (fever, weight loss), chest x-ray, MDR-TB, type of diagnosis, treatment with at least three active drugs, stratified by Center

^b^Omitted Rifampicin resistance in the multivariable model due to multicollinearity with MDR-TB

^c^Those not included in the categories “ART naïve at TB diagnosis” and”ART use at TB diagnosis” have previously received ART and interrupted their treatment

^d^Those with missing information for chest x-ray (n = 278), HBsAg (n = 215), Anti-HCV (n = 220) assumed negative

§ Index based on symptoms cough, fever, weight loss

### Sensitivity analysis

Of all 262 deaths within 24 months, 97 (37.0%) were directly TB-related. In an adjusted Cox proportional hazards model for TB-related mortality within 24 months follow-up delayed diagnosis had no effect on TB-related death (aHR 1.00; 95% CI 0.62–1.59; p = 0.988).

When stratifying delayed diagnosis by ≤ 3 months (n = 640) versus > 3 months (n = 100), at 24 months 247 (38.6%) deaths had occurred in the group of ≤ 3 months and 59 (59%) in the group of > 3 months. Kaplan–Maier survival analysis showed no difference in survival function (log-rank p = 0.777) (Additional file 1).

In an adjusted Cox proportional hazards model with 24 months of follow-up there was no effect of a delayed diagnosis for more than three months on all-cause mortality (aHR 1.00; 95% CI 0.7–1.45; p = 0.986).

## Discussion

In the present study, we found older age (≥ 50 years), IDU, ART naivety, disseminated TB and presenting with weight loss were associated with delayed diagnosis, while a previous TB diagnosis was associated with a lower risk of late diagnosis. Furthermore, a delayed TB diagnosis was associated with an increased risk for all-cause death in PLWH in Eastern Europe. Our study provides indication for a systematic screening and active case finding in PLWH at risk of delayed TB diagnosis.

### Factors associated with delayed diagnosis in PLWH

Our study is the first to present factors associated with delayed TB diagnosis in PLWH in Eastern Europe. Some of the factors identified in the present study, such as older age, being ART naive and IDU have been identified in other settings. In a South African cohort including only PLWH with pulmonary TB older age (> 40 years) and HIV-RNA viral load > 400 copies/ml were predictors for delayed diagnosis, while being on ART at time of diagnosis was associated with lower risk of delayed diagnosis [20]. A study including all drug-susceptible TB cases in PLWH from Colombia found age between 15–34, age ≥ 45 years, and receiving tuberculin skin tests as a part of the diagnostic work-up associated with delayed diagnosis [21]. In a Brazilian cohort of 242 PLWH with TB, factors associated with patient delay were the symptoms asthenia, chest pain, and the use of illicit drugs, including IDU [22]. In this current study, we identified a previous TB diagnosis as associated with lower risk of delayed diagnosis, which is in line with another cohort of PLWH from Brazil [23]. Weight loss and disseminated disease have not been previously described as associated with delayed diagnosis which could be due to better ascertainment of the symptom weight loss and disseminated disease presentation in our study or to uncontrolled circularity.

In patients diagnosed with TB (without HIV) in Kiev city long diagnostic delay was reported by individuals who were homeless, jobless or abused alcohol [24]. In Uzbekistan TB mono-infected patients self-medication, coughing, loss of weight, and visiting private and primary healthcare facilities were associated with delay [25]. Similar results were observed in Georgia where receipt of medication prior to TB diagnosis was associated with increased overall delayed diagnosis [26].

Disseminated TB was associated with delayed diagnosis in the present analysis. These diagnostic challenges have been described previously in PLWH as the extra-pulmonary or disseminated TB presentation makes it difficult to obtain suitable specimen for culture [27, 28].

### Effect of delayed diagnosis on survival in PLWH

In our study, those with a delayed diagnosis of at least 1 month had a 36% higher risk of dying within the first 2 years after the TB diagnosis. Compared to previous studies in other settings the effect seems less pronounced, and when adjusting for other patient characteristics, the result was no longer insignificant, although the effect size remains similar and the upper limit of the CI suggests it could be a quite large effect. Furthermore, there is a clear association between increased risk of death and male sex, low or missing CD4 cell count, prior AIDS, disseminated TB disease, weight loss and MDR-TB; all of which have been found and described in a previous study of the current cohort [17]. In a retrospective cohort of PLWH with pulmonary TB in China, a delayed diagnosis of more than 1 month from onset of symptoms increased the risk of death almost three-fold (aHR 2.60; 95% CI 1.42–4.78) [29]. In a Brazilian study, PLWH with TB who had previously started on ART, duration of TB symptoms over 3 months before diagnosis increased the risk of death six-fold (HR = 6.15, 95% CI 1.15–32.9) [14]. In Malawi, seeking care after more than 1 month or 3 months was associated with an increased risk of death of about 60% (aHR 1.6, 95% CI 0.9–2.8) and 100% (aHR 2.0, 95% CI 1.1–3.8), respectively [30]. On the contrary, a Thai study of 667 PLWH with TB found no effect of delayed TB diagnosis on survival [31].

Supplementary survival analyses stratified by follow-up time suggest that delayed diagnosis has no effect on survival during the first 2 months after TB treatment initiation, but seem to have an effect beyond 2 months after initiation of TB treatment, albeit insignificantly so, perhaps due to limited number of patients in the study. In our study, mortality was very high during the first 2 months, which leads us to speculate that those presenting with shorter symptom duration might be suffering from quickly progressing disease with worse early outcomes. That suggests that other factors were driving the mortality in the first 2 months diluting the effect of delayed diagnosis on survival in the early period. These could be factors such as disseminated disease, MDR-TB and an AIDS diagnosis which also are significant predictors of mortality in the stratified analysis 0–2 months (Additional file 1: Table S1).

### Potential implications for policy and research

It is very likely that better integration of clinical services and diagnostic capacity can decrease delays in diagnosing TB in PLWH. HIV and TB services in Eastern Europe are only provided in one place at about 36% of health care centers [32]. While evidence of the effect of integrated health care on delayed diagnosis is not available, studies in Sub-Saharan Africa have shown improved HIV and TB treatment outcomes when integrating HIV and TB care [33, 34].

Furthermore, strengthening diagnostic capacity for sputum-smear negative and extra-pulmonary TB (i.e., molecular diagnostic tests such as Xpert) are crucial as TB in PLWH often presents as extrapulmonary or disseminated TB and is commonly sputum-smear negative. There is evidence from Mozambique, where PLWH delayed for TB diagnosis due to initial negative sputum smear microscopy but consecutive positivity (Xpert TB test) had a 12-fold higher mortality, reiterating the importance of rapid diagnostics [16]. In Rwanda, expanded access to systematic Xpert testing and standardized treatment of Rifampicin-resistant TB in both HIV-seronegative and PLWH led to the reduction of diagnostic and treatment delays resulting in reduced mortality [35]. In Eastern Europe, Xpert was used as part of routine work-up at almost all (92%) clinics in 2018, which is a major improvement compared to 2013 (54%) when the present study was conducted, so we cannot rule out improvements to delayed diagnosis in the meantime [32]. A promising additional tool for rapid diagnosis and treatment initiation at the point of care for PLWH regardless of site of TB infection is the urine-based lipoarabinomannan (LAM) test [36]. Further prospective studies of this intervention and its effect on delayed diagnosis, in particular in an Eastern European context, are needed.

In addition to improving health care infrastructure, active case finding among high-risk populations, in our study those of older age, people who inject drugs and PLWH not on ART, may help reduce delayed diagnoses and improve linkage to health care [37]. In an active pulmonary TB case finding study among newly HIV diagnosed patients in Tbilisi, Georgia, 11.5% of patients were identified with pulmonary TB, highlighting the utility of an active case finding strategy [38]. Periodic screening for TB among PLWH using symptoms, chest x-ray, Xpert and/or urine-LAM point-of-care testing according to WHO guidelines could improve case finding [39]. However, to the best of our knowledge no evidence on the systematic implementation of these guidelines in Eastern Europe and Central Asia exists.

Our study has some limitations. First, the generalizability of our results to the background/general population may be impaired due to selection bias, as recruitment to our study is conditional on both TB and HIV being diagnosed. Those who were never diagnosed with TB or HIV due to early death without making contact to health care were not included. However, these patients presumably would have worse outcomes and a long diagnostic delay if included here, as reduced access to health care is likely associated with similar risk factors as delayed diagnosis, morbidity and mortality. Therefore, our results likely underestimate the effect of delayed diagnoses on mortality in the population. Secondly, Eastern Europe is heterogeneous, delay of diagnosis may vary within the region and in some countries, health care has improved since the period of data collection in 2013 [32]. As drug susceptibility testing was not widely available in Eastern Europe at the time of the study and those without testing results were assumed susceptible our study very likely underestimated MDR-TB and has led to increased mortality. Thirdly, we cannot rule out a certain degree of circularity in the analysis of risk factors for delayed diagnosis; e.g., disseminated disease or weight loss can be risk factors for delayed diagnosis due to an unexpected or hard to diagnosis disease presentation but they can also be a consequence of delayed diagnosis. Finally, for most patients in the present study the date of TB diagnosis and of treatment initiation were identical. Therefore, we were not able to calculate the health care delay, the time span between diagnosis and treatment initiation.

Regardless, the current study adds important knowledge to the field of health care for TB/HIV co-infection in Eastern Europe.

## Conclusions

We found that nearly two thirds of PLWH with TB in Eastern Europe had a delayed TB diagnosis, in particular those of older age, people who inject drugs, ART naïve, with disseminated disease, and presenting with weight loss. Patients with delayed TB diagnosis were subsequently at higher risk of death in unadjusted analysis. There is a clear need for optimization of the current TB diagnostic cascade and HIV care in PLWH in Eastern Europe to reduce delayed diagnoses to reduce morbidity and improve treatment outcomes. While health care planners and policy makers should ensure strengthened diagnostic capacity, clinicians should pay special attention to patient groups identified as being at higher risk for delayed TB diagnosis, and further research should focus on developing more effective diagnostic interventions to reduce delayed diagnoses.

### Supplementary Information

**Additional file 1: Table S1.** Cox-regression model for survival in Eastern Europe, crude and adjusted hazard ratios for follow-up 0–2 months. **Table S2.** Cox-regression model for survival in Eastern Europe, crude and adjusted hazard ratios for follow-up 2–24 months. **Figure S1.** Kaplan–Meier survival estimates by diagnostic delay (≤ 3 months versus > 3 months)

## Data Availability

The datasets used and/or analyzed during the current study are available from the corresponding author on reasonable request.

## Appendix TB:HIV Study Group

www.chip.dk/Research/Studies/TBHIV/TBHIV-Study-Group

The Study Group consists of the following 62 participating sites in 19 countries (listed with countries, names of participating centers).

A. Vassilenko^14^, D. Klimuk^15^, O. Kondratenko^15^, A. Zalutskaya^15^, V. Bondarenko^16^, V. Mitsura^16^, E. Kozorez^16^, O. Tumash^16^, O. Suetnov^17^, D. Paduto^17^, V. Iljina^18^, T. Kummik^18^, K. Mshvidobadze^19^, N. Lanchava^19^, L. Goginashvili^20^, L. Mikiashvili^20^, N. Bablishvili^20^, B. Rozentale^21^, I. Zeltina^21^, I. Janushkevich^21^, I. Caplinskiene^22^, S. Caplinskas^22^, Z. Kancauskiene^22^, A. Wiercinska-Drapalo^23^, M. Thompson^24^, J. Kozlowska^24^, A. Grezesczuk^24^, M. Bura^25^ B. Knysz^26^, M. Inglot^26^, A. Garlicki^27^, J. Loster^27^, D. Duiculescu^28^, A. Rakhmanova^29^, O. Panteleev^29^, A. Yakovlev^29^, A. Kozlov^29^, A. Tyukalova^29^, Y. Vlasova^29, 30^, T. Trofimov^31^, G. Kyselyova^32^, N. Obel^33^, J. Gerstoft^33^, G. Kronborg^34^, MC Payen^35^, K. Kabeya^35^, C. Necsoi^35^, F. Dabis^36^, A. Tsaranazy^36^, C. Cazanave^37^, H. Furrer^38^, M. Sagette^38^, M. Rickenbach^38^, D. Sculier^39^, A. Calmy^39^, M. Cavassini^40^, A. Bruno^41^, E. Bernasconi^41^, M. Hoffmann^42^, P. Vernazza^42^, J. Fehr^43^, R. Weber^43^, R. Miller^44^, N. Vora^44^, G. Cooke^45^, S. Mullaney^45^, E. Wilkins^46^, V. George^46^, P. Collini^47^, D. Dockrell^47^, F. Post^48^, L. Campbell^48^, R. Brum^48^, E. Mabonga^48^, P. Saigal^48^, S. Kegg^49^, J. Ainsworth^50^, A. Waters^50^ J. Dhar^51^, K. Ellis^51^, E. Girardi^52^, A Rianda^52^, V. Galati^52^, C. Pinnetti^52^, C. Tommasi^52^, G. Lapadula^53^, A. Di Biagio^54^, A. Parisini^54^, S. Carbonara^55^, G. Angarano^55^, M. Purgatorio^55^, A. Matteelli^56^, A. Apostoli^56^, JM. Miro^57^, C. Manzardo^57^, C. Ligero^57^, J. Gonzalez^57^, Jose A. Martinez-Martinez^57^, F. Sanchez^58^, H. Knobel^58^, M. Salvadó^58^, J.L. Lopez-Colomes^58^, X. Martínez-Lacasa^59^, E. Cuchí^59^, V. Falcó^60^, A. Curran^60^, M.T. Tortola^60^, I. Ocaña^60^, R. Vidal^60^, MA. Sambeat^61^, V. Pomar^61^, P. Coll^61^, D. Pozamczer^62^, M. Saumoy^62^, F. Alcaide^62^, J. Caylà^63^, A. Moreno^63^, J.P. Millet^63^, A. Orcau^63^, L. Fina^63^, A. Romero^63^, L.L. Roldan^63^, JA. Iribarren^64^, M. Ibarguren^64^, S. Moreno^65^, A. González^65^, P. Miralles^66^, T. Aldámiz-Echevarría^66^, M. Losso^67^, J. Toibaro^67^, L. Gambardella^67^, J. Toibaro^68^, L. Moreno Macias^68^, E. Warley^69^, S. Tavella^69^, O. Garcia Messina^70^, O. Gear^70^, H. Laplume^71^, C. Marson^72^, J. Contarelia^73^, M. Michaan^73^, P. Scapellato^74^, B. Bartoletti^75^, D. Palmero^75^, C. Elias^76^, C. Cortes^77^, B. Crabtree^78^, JL Mosqueda Gomez^79^, J. A. Villanueva^80^; LA Gonzalez Hernandez^80^ and F. Badial^80^.

14 Belarusian State Medical University, Department of Infectious Disease.

15 Republican Research and Practical Centre for Pulmonology.

16 Gomel State Medical University (Gomel).

17 Gomel Region Centre for Hygiene: O. Suetnov (PI) and D. Paduto.

18 East Viru Central Hospital (Kohtla-Jarve), Estonia.

19 Infectious Diseases, AIDS and Clinical Immunology Research Center (Tiblisi).

20 National Center for Tuberculosis and Lung Diseases of Georgia (Tibilisi).

21 Infectology Centre of Latvia (Riga).

22 Centre for Communicable Diseases and AIDS (Vilnius).

23 Wojewodski Szpital Zakanzy/Medical University of Warsaw (Warszawa).

24 Wojewodski Szpital Specjalistyczny/Medical University Teaching Hospital (Bialystok).

25 Jozef Strus Multidisciplinary City Hospital (Poznan).

26 Wroclaw University School of Medicine (Wroclaw).

27 Jagiellonian University Medical College (Krakow).

28 Dr. Victor Babes Hospital (Bucharest).

29 Botkin Hospital of Infectious Diseases (St. Petersburg).

30 City TB Hospital No. 2 (St. Petersburg):

31 Center for Prevention and Control of AIDS (Veliky, Novgorod).

32 Crimean Republican AIDS Centre (Simferopol).

33 Rigshospitalet (Cph).

34 Hvidovre University Hospital.

35 CHU Saint-Pierre (Brussels).

36 Aquitaine Cohort.

37 Bordeaux University Hospital.

38 University Hospital Bern.

39 Hopital Cantonal Universitaire, Geneve.

40 Centre Hospitalaire Universitaire Vaudois, Lausanne.

41 Hospital of Lugano.

42 Cantonal Hospital St. Gallen.

43 University Hospital Zurich.

44 Mortimer Market Centre (London).

45 St. Mary’s Hospital.

46 North Manchester General Hospital.

47 Sheffield Teaching Hospitals.

48 King’s College Hospital (London).

49 Queen Elizabeth Hospital.

50 North Middelsex University Hospital.

51 Leicester Royal Infirmary.

52 IRCCS—Ospedale L. Spallanzani (Rome).

53 AO San Gerardo (Monza).

54 IRCCS AOU San Martino – IST di Genoa (Genova).

55 Clinic of Infectious Diseases, University of Bari (Bari).

56 University of Brescia Spedali Civili.

57 Hospital Clinic of Barcelona.

58 Hospital del Mar.

59 Mutua de Terrassa.

60 Hospital Universitari Vall d’Hebrón:

61 Hospital Universitari de la Santa Creu i Sant Pau.

62 Hospital Universitari de Bellvitge.

63 Agencia de Salud Pública de Barcelona.

64 Hospital Universitaria Donostia (San Sebastian).

65 Hospital Ramon y Cajal (Madrid).

66 Hospital Universitaria’Gregorio Marañon’’ (Madrid).

67 The CICAL Cohort, Argentina.

68 Hospital J. M. Ramos Mejía (Buenos Aires).

69 Hospital Paroissien (BA).

70 Hospital Piñero (BA).

71 Hospital Nacional Profesor Alejandro Posadas:

72 Hospital Rawson (Cordoba).

73 Hospital San Juan de Dios (La Plata).

74 Hospital General de Agudos Donación F. Santojani.

75 Hospital Francisco Javier Muñiz (BA).

76 Hospital Jujuy.

77 Fundación Arriaran (Santiago).

78 INNcMZS (México DF).

79 Hospital General Regional de Leon- CAPACITS.

80 Hospital Civil de Guadalajara.

## Abbreviations

TB: Tuberculosis
HIV: Human immunodeficiency virus
PLWH: People living with HIV
AIDS: Acquired immunodeficiency syndrome
IEC: Independent Ethics Committee
IRB: Institutional Review Board
RNA: Ribonucleotide acid
ART: Antiretroviral therapy
HBsAg: Hepatitis B surface antigen
HCV: Hepatitis C virus
MDR: Multi-drug resistant
PCR: Polymerase chain reaction
Mtb: Mycobacterium tuberculosis
CSF: Cerebrospinal fluid
KM: Kaplan–Meier
HR: Hazard ratio
CI: Confidence interval
IDU: Injecting drug use
OR: Odds ratio
PYFU: Person years of follow-up
LAM: Lipoarabinomannan
