# Supplementary material for: Delayed diagnosis of tuberculosis in persons living with HIV in Eastern Europe: associated factors and effect on mortality—a multicentre prospective cohort study
Source: BMC Infect Dis. 2021 Oct 6;21:1038. doi: 10.1186/s12879-021-06745-w (PMC8496077; doi:10.1186/s12879-021-06745-w)

Supplementary File

Table S1. Cox-regression model for survival in Eastern Europe, crude and adjusted hazard ratios for follow-up 0–2 months

|  | **Total (n = 740)** | **Crude HR**  **(95 % CI)** | **p-value** | **Adjusted HR (95% CI)*** | **p-value** |
| --- | --- | --- | --- | --- | --- |
| Diagnostic Delay | >1 month (n = 480) | 1.11 (0.74 – 1.66) | 0.625 | 1.09 (0.69 – 1.71) | 0.723 |
| Gender | Male (n = 563) | 1.56 (1.04 – 2.34) | 0.033 | 1.45 (0.94 – 2.25) | 0.092 |
| Age | Age 18 – 49 (n = 694) | Ref | - |  |  |
|  | Age ≥50 (n = 46) | 0.86 (0.38 – 1.95) | 0.719 |  |  |
| Exposure Group (HIV) | MSM (n = 10) | 0.69 (0.1 – 4.98) | 0.717 |  |  |
|  | IDU (n = 422) | 1.08 (0.74 – 1.59) | 0.681 |  |  |
|  | Heterosexual (n = 183) | 0.93 (0.60 – 1.46) | 0.768 |  |  |
| Treatment history (HIV) | Known HIV positive (n = 668) | 1.37 (0.67 – 2.82) | 0.390 |  |  |
|  | Naïve at TB diagnosis (n = 558) | 0.98 (0.64 – 1.52) | 0.939 |  |  |
|  | ART at baseline (n = 132) | 1.20 (0.75 – 1.92) | 0.442 |  |  |
|  | Cotrimoxazole at baseline (n = 273) | 1.05 (0.71 – 1.55) | 0.810 |  |  |
| CD4-cell count/mm3 ° | 0 – 199/mm3 (n = 440) | 2.49 (1.38 – 4.49) | 0.002 | 1.66 (0.89 – 3.10) | 0.114 |
|  | ≥200/mm3 ( n = 185) | Ref | Ref | Ref | Ref |
|  | Missing CD4 (n = 115) | 2.56 (1.27 – 5.15) | 0.008 | 2.66 (1.27 – 5.56) | 0.009 |
| Prior AIDS | Yes (n = 177) | 3.20 (2.19 – 4.68) | 0.001 | 2.88 (1.87 – 4.43) | 0.001 |
| Previous TB | Yes (n = 99) | 1.08 (0.63 – 1.88) | 0.761 |  |  |
| TB risk factor | Alcohol Abuse (n = 186) | 1.67 (1.12 – 2.49) | 0.011 | 1.38 (0.88 – 2.16) | 0.155 |
|  | Recent TB in family (n = 53) | 0.93 (0.43 – 2.01) | 0.857 |  |  |
|  | Prison within the last 2 years (n = 143) | 0.73 (0.43 – 1.24) | 0.247 |  |  |
| Clinical Presentation of TB | Pulmonary (n = 239) | Ref | Ref | Ref | Ref |
|  | Extrapulmonary (n = 54) | 1.34 (0.50 – 3.64) | 0.562 | 1.32 (0.46 – 3.78) | 0.605 |
|  | Disseminated (n = 447) | 2.86 (1.70 – 4.82) | 0.001 | 2.64 (1.49 – 4.69) | 0.001 |
| TB symptoms at diagnosis | Cough (n = 501) | 1.44 (0.93 – 2.22) | 0.103 | 1.32 (0.82 – 2.13) | 0.252 |
|  | Fever (n = 636) | 1.44 (0.77 – 2.68) | 0.253 |  |  |
|  | Weight loss (n = 445) | 2.53 (1.59 – 4.01) | 0.001 | 2.11 (1.27 – 3.50) | 0.004 |
| Number of symptoms§ | None (n = 20) | Ref | Ref |  |  |
|  | One Symptom (n = 133) | 0.56 (0.12 – 2.66) | 0.469 |  |  |
|  | Two Symptoms (n = 312) | 1.21 (0.29 – 5.02) | 0.790 |  |  |
|  | Three Symptoms (n = 275) | 2.11 (0.52 – 8.65) | 0.299 |  |  |
| Screening Chest X-Ray ß | Yes (n = 338) | 1.38 (0.94 – 2.01) | 0.100 | 1.80 (1.14 – 2.86) | 0.013 |
| Rifampicin Resistance | Yes (n = 95) | 1.66 (1.03 – 2.67) | 0.038 | Omitted** |  |
| MDR-TB | Yes (n = 88) | 1.70 (1.04 – 2.76) | 0.033 | 2.98 (1.48 – 6.00) | 0.002 |
| Diagnosis | Definitive (n = 333) | Ref | Ref | Ref | Ref |
|  | Probable (n = 76) | 1.78 (0.99 – 3.22) | 0.056 | 1.56 (0.71 – 3.43) | 0.264 |
|  | Presumptive (n = 331) | 1.40 (0.92 – 2.11) | 0.112 | 1.72 (0.91 – 3.25) | 0.096 |
| Hepatis B | HBsAg positive (n = 44)ß | 1.12 (0.52 – 2.42) | 0.766 |  |  |
| Hepatitis C | Anti-HCV positive (n = 405)ß | 1.57 (1.06 – 2.33) | 0.026 |  |  |
| Drug-susceptibility testing | At baseline (n = 268) | 0.77 (0.52 – 1.16) | 0.219 |  |  |
| Treatment with RHZ | At baseline (n = 591) | 0.71 (0.46 – 1.10) | 0.123 |  |  |
| Treatment with at least three active drugs | At baseline (n = 132) | 0.55 (0.30 – 1.00) | 0.049 | 0.96 (0.44 – 2.09) | 0.917 |

HR – Hazard Ratio; CI – Confidence Interval; MSM – Men having sex with men; IDU – Injecting Drug Use

*adjusted for diagnostic delay, gender, CD4 cell count, prior aids, tb risk factor alcohol, clinical presentation (all), Tb symptoms (cough, weight loss), chest x-ray, MDR-TB, type of diagnosis, treatment with at least three active drugs, stratified by Center

**Omitted Rifampicin resistance in the multivariable model due to multicollinearity with MDR-TB

ß Those with missing information for chest x-ray (n = 278), HBsAg (n = 215), Anti-HCV (n = 220) assumed negative

Table S2. Cox-regression model for survival in Eastern Europe, crude and adjusted hazard ratios for follow-up 2-24 months

|  | **Total (n = 610)** | **Crude HR**  **(95 % CI)** | **p-value** | **Adjusted HR (95% CI)*** | **p-value** |
| --- | --- | --- | --- | --- | --- |
| Diagnostic Delay | >1 month (n = 393) | 1.56 (1.10 – 2.21) | 0.012 | 1.36 (0.93 – 1.99) | 0.119 |
| Gender | Male (n = 472) | 1.20 (0.84 – 1.73) | 0.322 |  |  |
| Age | Age 18 – 49 (n = 570) | Ref | - |  |  |
|  | Age ≥50 (n = 40) | 0.96 (0.50 – 1.82) | 0.894 |  |  |
| Exposure Group (HIV) | MSM (n = 9) | 0.37 (0.05 – 2.67) | 0.326 |  |  |
|  | IDU (n = 342) | 1.26 (0.92 – 1.75) | 0.153 |  |  |
|  | Heterosexual (n = 152) | 0.79 (0.53 – 1.15) | 0.219 |  |  |
| Treatment history (HIV) | Known HIV positive (n = 547) | 1.69 (0.89 – 3.20) | 0.110 |  |  |
|  | Naïve at TB diagnosis (n = 458) | 1.09 (0.76 – 1.56) | 0.657 |  |  |
|  | ART at baseline (n = 108) | 0.82 (0.54 – 1.24) | 0.345 |  |  |
|  | Cotrimoxazole at baseline (n = 273) | 1.36 (0.99 – 1.87) | 0.060 | 1.00 (0.68 – 1.47) | 0.993 |
| CD4-cell count/mm3 ° | 0 – 199/mm3 (n = 351) | 2.01 (1.30 – 3.11) | 0.002 | 1.95 (1.20 – 3.18) | 0.007 |
|  | ≥200/mm3 ( n = 164) | Ref | Ref | Ref | Ref |
|  | Missing CD4 (n = 95) | 1.96 (1.13 – 3.39) | 0.017 | 1.78 (1.02 – 3.12) | 0.044 |
| Prior AIDS | Yes (n = 126) | 1.21 (0.84 – 1.74) | 0.315 |  |  |
| Previous TB | Yes (n = 79) | 0.90 (0.55 – 1.45) | 0.657 |  |  |
| TB risk factor | Alcohol Abuse (n = 142) | 1.10 (0.76 – 1.59) | 0.603 |  |  |
|  | Recent TB in family (n = 43) | 0.78 (0.38 – 1.59) | 0.494 |  |  |
|  | Prison within the last 2 years (n = 119) | 1.44 (0.99 – 2.09) | 0.057 | 1.40 (0.94 – 2.08) | 0.098 |
| Clinical Presentation of TB | Pulmonary (n = 214) | Ref | Ref | Ref | Ref |
|  | Extrapulmonary (n = 46) | 0.81 (0.34 – 1.92) | 0.635 | 0.71 (0.29 – 1.77) | 0.463 |
|  | Disseminated (n = 350) | 2.05 (1.42 – 2.96) | 0.001 | 1.66 (1.09 – 2.53) | 0.019 |
| TB symptoms at diagnosis | Cough (n = 402) | 0.74 (0.54 – 1.02) | 0.066 | 0.74 (0.51 – 1.05) | 0.092 |
|  | Fever (n = 521) | 1.70 (1.01 – 2.85) | 0.045 | 1.49 (0.85 – 2.62) | 0.168 |
|  | Weight loss (n = 352) | 1.35 (0.97 – 1.87) | 0.071 | 1.16 (0.81 – 1.66) | 0.426 |
| Number of symptoms§ | None (n = 17) | Ref | Ref |  |  |
|  | One Symptom (n = 122) | 1.05 (0.37 – 2.99) | 0.922 |  |  |
|  | Two Symptoms (n = 260) | 1.08 (0.39 – 2.97) | 0.880 |  |  |
|  | Three Symptoms (n = 211) | 1.28 (0.47 – 3.51) | 0.638 |  |  |
| Screening Chest X-Ray ß | Yes (n = 271) | 1.25 (0.91 – 1.71) | 0.163 |  |  |
| Rifampicin Resistance | Yes (n = 74) | 1.45 (0.96 – 2.20) | 0.079 | Omitted** |  |
| MDR-TB | Yes (n = 68) | 1.55 (1.02 – 2.37) | 0.041 | 1.62 (1.00 – 2.62) | 0.051 |
| Diagnosis | Definitive (n = 291) | Ref | Ref | Ref | Ref |
|  | Probable (n = 59) | 1.24 (0.73 – 2.09) | 0.056 | 0.99 (0.55 – 1.79) | 0.976 |
|  | Presumptive (n = 260) | 0.85 (0.60 – 1.19) | 0.334 | 0.84 (0.57 – 1.23) | 0.368 |
| Hepatis B | HBsAg positive (n = 37)ß | 0.83 (0.41 – 1.70) | 0.766 |  |  |
| Hepatitis C | Anti-HCV positive (n = 319)ß | 1.01 (0.73 – 1.38) | 0.974 |  |  |
| Drug-susceptibility testing | At baseline (n = 268) | 0.88 (0.64 – 1.22) | 0.456 |  |  |
| Treatment with RHZ | At baseline (n = 591) | 0.90 (0.60 – 1.35) | 0.601 |  |  |
| Treatment with at least three active drugs | At baseline (n = 132) | 0.71 (0.46 – 1.09) | 0.115 |  |  |

HR – Hazard Ratio; CI – Confidence Interval; MSM – Men having sex with men; IDU – Injecting Drug Use

*adjusted for diagnostic delay, Cotrimoxazol at baseline, CD4 cell count, risk factor prison, clinical presentation (all), Tb symptoms (cough, fever, weight loss), MDR-TB, type of diagnosis, stratified by Center

**Omitted Rifampicin resistance in the multivariable model due to multicollinearity with MDR-TB

ß Those with missing information for chest x-ray (n = 278), HBsAg (n = 215), Anti-HCV (n = 220) assumed negative

Supplementary Figure 1. Kaplan-Meier survival estimates by diagnostic delay (≤ 3months versus >3 months)
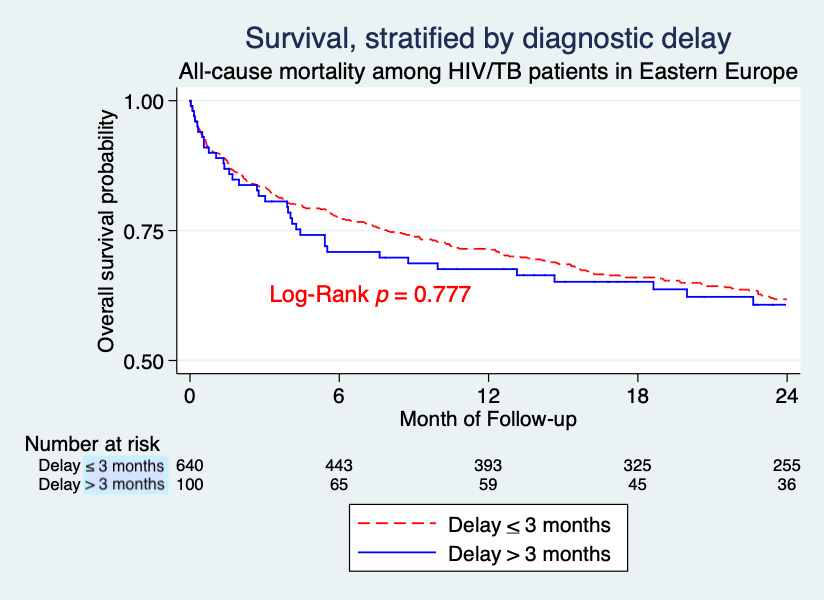

Supplement: Supplementary file 1 — Additional file 1: Table S1. Cox-regression model for survival in Eastern Europe, crude and adjusted hazard ratios for follow-up 0–2 months. Table S2. Cox-regression model for survival in Eastern Europe, crude and adjusted hazard ratios for follow-up 2–24 months. Figure S1. Kaplan–Meier survival estimates by diagnostic delay (≤ 3 months versus > 3 months) [file 12879_2021_6745_MOESM1_ESM.docx]
